# Biochar as Sustainable Filler of Recycled Polylactic Acid (PLA): A New Generation of Processable Biocomposites

**DOI:** 10.3390/polym16233347

**Published:** 2024-11-29

**Authors:** Chiara Botta, Corinna Maria Grottola, Davide Amato, Maria Rosaria Acocella

**Affiliations:** 1 Agricola Imballaggi S.r.l., 84044 Albanella, Italy; cb@agricolaimballaggi.it; 2 Institute of Sciences and Technologies for Sustainable Energy and Mobility (STEMS), National Research Council (CNR), 80125 Naples, Italy; corinnamaria.grottola@stems.cnr.it (C.M.G.); davide.amato@stems.cnr.it (D.A.); 3 Department of Chemistry and Biology “A. Zambelli”, INSTM Research Unit, University of Salerno, Via Giovanni Paolo II, 132, 84084 Fisciano, Italy

**Keywords:** biochar, recycled PLA, nucleation effect, composites, stabilization effect, aging treatment

## Abstract

As an alternative to fossil-based polymers, polylactide acid (PLA) has stimulated a significant research effort in the past few years due to the demand for environmentally friendly products. Even though PLA is considered a sustainable or bio-based material, the long-term degradation in ambient conditions increases the volume of waste at end-of-life. To overcome this issue, PLA waste can be recycled to produce new manufactures; however, this approach does not always ensure the same mechanical properties as the original PLA. This study shows for the first time that adding biochar, a carbon material derived from biomass pyrolysis, enhances the processability and stability of composite recycled PLA. Composites are provided in 1, 2.5, and 5 wt% of the biochar filler, resulting in good processability, a higher modulus of up to 20%, and a higher stability to degradation in the presence of UV aging treatment with respect to the raw material. Additionally, DSC analysis shows a significant nucleation effect induced by the biochar that achieves 30% crystallinity from an essentially amorphous PLA.

## 1. Introduction

PLA is a well-known biodegradable polymer that, as an alternative to polymers of fossil sources, has stimulated a growing research effort in the last years due to the increasing demand for environmentally sustainable products [1].

Due to its commercial availability, PLA has been used for many applications, such as the medical sector, fibers and textiles, packaging and service ware, environmental remediation, and agriculture [2].

Although PLA has a leading position for replacing petrochemical-derived polymers due to its relevant physical–chemical properties, as well as its bio-sourced origin and compostability, its application could be limited by some drawbacks, such as high hydrophilicity, low gas barrier properties, sensitivity to ultraviolet (UV) exposure (e.g., sunlight), and brittleness [3].

To overcome these problems, the preparation of composites and nanocomposites with fillers of different natures was provided, showing improved mechanical, thermal, rheological, optical, and photodegradation properties [4,5,6,7,8].

Recently, the ability of carbon materials, such as high surface area graphite and carbon black, to thermally stabilize the PLA during the melting processing was reported to prevent possible degradation of the molecular weight and save the mechanical properties of the biopolymer [9]. Additionally, the functionalization of oxidized carbon black by alkylation with long alkyl chains was effective in providing a fast kinetic crystallization, preserving the molecular weight and, therefore, the mechanical properties of the biopolymer [10].

Although graphite-based materials have shown outstanding ability in generating PLA composites, the sustainability requirements point out the fossil nature of the carbon materials used and highlight a necessary change of route for respecting the environmental requisites.

An interesting alternative to carbon from fossil sources is biochar, a porous carbonaceous material produced through pyrolysis, which involves heating biomass in an inert environment [11]. The physical and chemical properties of biochar that determine its applications are correlated to the pyrolysis operating conditions and feedstock composition [12,13].

During pyrolysis at a low heating rate and long residence time, the decomposition of the biomass releases oxygenated volatile compounds, thus leaving a solid matrix, i.e., the biochar, with a well-developed porosity. At the same time, as temperature increases, the functional groups containing oxygen and hydrogen decrease on the surface of the biochar, whereas the carbon content increases, as well as biochar aromaticity. In the typical temperature range of pyrolysis for biochar production, the inorganic species are only slightly released, resulting in an increase of the ash content in the biochar as pyrolysis temperature increases, which is responsible for the basic pH observed for biochar produced at high temperatures.

Traditionally, biochar has been used for soil amendment due to its ability to improve soil fertility, water retention, and soil structure [11]; however, it has broadened its application across diverse fields, from the environment to the electronic sector [14].

Biochar is used in water and soil purification systems to remove heavy metals, pesticides, and organic pollutants due to its high adsorption capacity and ability to stabilize toxic elements [15,16]. It has also been studied as a filler in concrete, asphalt, and building materials to enhance thermal insulation and reduce construction’s carbon footprint [17], as a conductive material for energy storage and electronics, and as a conductive filler in polymer-based composites [18], supercapacitors, and electrodes [19].

It has been recently reported that the use of biochar derived from spent ground coffee showed a good level of filler dispersion within the PLA matrix, notwithstanding the occurrence of a remarkable decrease of the PLA molar mass during the processing at high temperatures, possibly due to residual hydroxyl functionalities present on the biochar surface [20,21,22].

Therefore, the careful selection of the pyrolysis temperature and the nature and distribution of the functional groups on the carbon surface can prevent degradation and help the PLA increase its mechanical properties [22].

Moreover, different feedstocks can deeply affect the PLA stability during the processing, being characterized by different amounts and nature of ashes. Specifically, the presence of potassium content and its progressive increase can accelerate PLA decomposition [23].

Although several studies have been published on using biochar in PLA, they all used commercial pellets of high molecular weight known as virgin material [22,24,25,26,27,28].

As a virgin material, PLA shows clear advantages in climate protection and fossil resource conservation compared to fossil-based plastics in life-cycle assessment (LCA) studies. In contrast, negative environmental impacts are often calculated for PLA source biomass production. The impact of land use and water consumption on the production of the starting monomer determines high costs and makes switching from conventional plastics to bio-based plastics not so straightforward [29].

Furthermore, PLA’s biodegradability cannot ensure its degradation into the soil at ambient temperature within a reasonable timeframe, resulting in the need for new bio-based plastic waste management systems to handle their end-of-life.

Therefore, the mechanical recycling of PLA could be an interesting approach to handling the PLA’s end-of-life and obtaining a material suitable for reuse. However, the thermal treatment due to the melt processing negatively alters the mechanical properties of the regenerated material (e.g., molecular weight reduction) and results in downcycling of the original polymer, thus reducing the viscoelastic and mechanical performance of the recyclates and limiting the number of reuse cycles

Additionally, the final properties of the recycled PLA are hardly determined by the source of the waste. To limit a possible heterogeneous composition due to the biopolymer’s different applications and properties, industrial PLA waste could be the successful approach for a more sustainable route.

In order to confer recycled PLA with improved properties, fillers are used to form PLA-based composites, which is one of the preferred routes. Although silk fibers have been reported to favor nucleation and reduce viscosity during multiple reprocessing [30], the use of carbon-based fillers is more attractive given their low cost, availability, and biodegradability.

In this study, recycled PLA, derived by melt extrusion of industrial PLA waste, was for the first time extruded with biochar for providing a new PLA composite by using 1, 2.5, and 5% filler loading. The composites were prepared by melt extrusion, and their chemical and mechanical properties were characterized. The results show that biochar has a good ability to prevent PLA degradation during processing under UV irradiation and ensure interesting mechanical properties.

## 2. Materials and Method

### 2.1. Materials

Biochar (B) was produced from the slow pyrolysis of Poplar (P). Pyrolysis tests were carried out at the final temperature of 450 °C in a fixed-bed reactor at a low heating rate (HR) of 10 °C/min, with a constant nitrogen flow (N_2_ = 12 Nl/min).

The thermal behavior of the feedstock was explored in thermogravimetric analysis (TGA) using Q500 TA Instruments (New Castle, DE, USA) in an inert environment (N2) from 10 to 800 °C at an HR of 10 °C. The feedstock and the corresponding biochar were characterized through proximate analysis using the ASTM E870 procedure. Ultimate analysis was performed using an elemental analyzer CHN 2000 LECO (Milan, Italy) analyzer, based on the CEN/TS 15104 procedure. The oxygen was evaluated by difference, considering the C, H, N, and ash content, calculated on a dry basis (db). Ash composition was performed by dissolving the samples of P and B via microwave-assisted acid digestion based on methods 3051 and 3052. After the digestion, the samples were then analyzed by inductively coupled plasma mass spectrometry (ICP/MS) using an Agilent 7500CE instrument (Santa Clara, CA, USA). The detection limit for all the inorganic species is 0.1 mg kg^−1^. Biomass and biochar pH was evaluated following the ASTM D4972-13 standard procedure, with a digital pH meter (827 pH LAB, Metrohm (Rome, Italy)), by measuring in deionized water using a 1:20 wt/wt ratio. The pHpzc was determined following the procedure reported by Mahmood et al. (2011) [31] The porosity of B was obtained using an Autosorb-1 (Quantachrome) instrument (Boynton Beach, FL, USA) with N_2_ at −196 °C as the adsorbate gas. Before the porosity analysis, the sample was degassed at 200 °C for 6 h under vacuum conditions. The surface area was evaluated using the Brunauer–Emmett–Teller equation (BET). Three replicates were conducted for all the analyses.

(r-PLA) was purchased by Filippo Russo Company as a regenerated one from PLA industrial waste without any additive or chain extender, using a mechanical treatment of waste during preparation and granulation produced by extrusion.

### 2.2. Processing

r-PLA and r-PLA-based composites containing 1, 2.5, 5 wt.% of BC were processed by extrusion using a co-rotating twin-screw extruder TBT SIEITECH Instruments & Equipment Co., Ltd. (Nanjing, China) with a screw diameter of 20 mm, L/D ratio equal to 1:40 operating at 180 °C, 150 rpm. Before the processing, r-PLA and BC were dried for 24 h at 60 °C.

For each formulation, ten dog bone-shaped tensile test specimens were prepared. Specimens for the various characterizations were obtained through an injection molding press (Sandretto Metalmeccanica—Serienove125) at 190 °C and 900 bar pressure. Prior to the processing, PLA and BC were vacuum-dried overnight at 70 °C.

The mechanical properties of the composites were evaluated using a dynamometer LLOYD INSTRUMENT LRX (UK). Tensile strength tests were performed, and a load was applied at a rate of 1 mm/min crosshead movement with a 25 kN load cell.

In order to evaluate the flexural Young Modulus of the specimens, a Two-point loading test was conducted, and a load was applied at a rate of 1 mm/min crossheat movement with a 25 kN load cell.

### 2.3. Characterization Techniques

TGA Thermogravimetric analyses (TGA) were performed using a PyrisTGA 4000 (Perkin Elmer, Shelton, CT, USA). Samples (about 20 mg) were placed in alumina pans, and runs were carried out in the range of 40–500 °C, with a heating rate of 20 °C/min, under N_2_ flow (30 mL/min).

Differential Scanning Calorimetry (DSC) analysis was carried out using a DSC3+ (Mettler Toledo (Greifensee, Zurich) using samples (from 5 to 25 mg) placed in aluminum pans sealed with aluminum lids. The samples were subjected to the following heat–cool–heat cycles in a nitrogen gas flow of 50 mL/min: • First heating from 25 to 200 °C at 50 °C/min. • Cooling from 200 to 25 °C at 10 °C/min. • Second heating from 25 to 200 °C at 10 °C/min. The thermal parameters, i.e., glass transition temperature (T_g_), cold crystallization temperature (T_cc_), melting temperature (T_m_), crystallization temperature (T_c_), and enthalpies, were determined.

The degree of crystallinity (*X*_c_) of the neat PLA and PLA composites was calculated using the equation:

*X*_c_ = (Δ*H*_m_ − Δ*H*_cc_)·(1/Δ*H*^0^*m* ∗ *w*) (1)

where Δ*H*_m_ is the experimental heat of fusion determined from DSC, Δ*H*^0^ is the theoretical heat of fusion of the 100% crystalline PLA (93.7 J/g), and *w* is the weight fraction of PLA in the composites [9].

Aging treatment: PLA has been submitted at artificial weathering with filtered xenon-arc radiation in order to obtain the degree of change of selected properties after a specific radiant exposure. The aging treatment was carried out using a Xenon Light Accelerated Weathering Tester BGD 866/A (Biuged Laboratory Instruments (Guangzhou) CO., LTD, Guangzhou, China). The samples were subjected to light radiation from 0 to 130 h at 0.35 W/m^2^ of irradiance and a BPT (Black Panel Temperature) of 63 °C.

Melt flow index: The melt flow index (MFI) of PLA is investigated using a Melt Flow Indexer 2322 (AMSE s.r.l., Torino, Italy). The samples were subjected to the conditions of 190 °C/2.16 kg by the D1238 ASTM procedure.

## 3. Results and Discussion

### 3.1. Characterization of the Carbon Material

The solid residue from Populous nigra treated at 450 °C and slow heating rate (10 °C min^−1^) in an inert environment (N_2_ = 12 Nl/min) was used in this study. The raw material was fully characterized by elemental analysis (EA), FTIR, ICP, WAXD, and BET.

The carbon content, the fixed carbon, and pH (Table 1) are in agreement with those reported in the literature [32]. The amount and composition of ash varies with the type of biomass used. In the case of Poplar, most of the metals are alkali and alkaline earth metals, as reported in Table 2.

**Figure 1:**
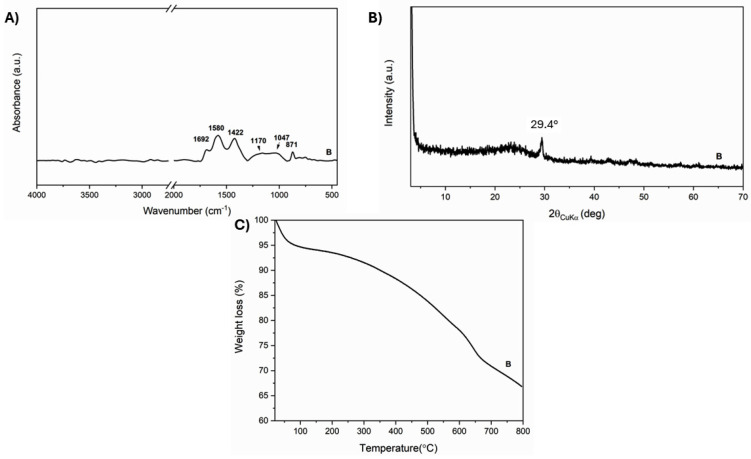
FTIR (**A**), X-Ray diffraction pattern (**B**), TGA (**C**) of biochar B.

The FTIR of B reveals broad bands at 1692, 1580, and 1422 cm^−1^ due to the presence of the carbonyl group (essentially ketones and lactones), C=C vibration, and CH_2_ bending, respectively. Additionally, alcohol and ether group vibrations are evident at 1170, 1047, and 871 cm^−1^ (Figure 1 A). [18] The TGA profile agrees with the O/C ratio and the residual ashes.

More interesting is the XRD profile (Figure 1B), which shows the typical broad reflection centered roughly on 23° of the amorphous carbon materials with a sharp peak at 2θ 29.4° due to the presence of the significant amount of calcium carbonate [33].

### 3.2. Preparation and Characterization of the PLA-Based Composites

The preparation of the PLA composites was performed by using regenerated PLA (r-PLA) derived by processing industrial PLA waste and 1, 2.5, and 5 wt% of biochar (B) by melt extrusion process at T = 180 °C, providing e-1B-PLA, e-2.5B-PLA, and e-5B-PLA, respectively.

To evaluate the stability of the r-PLA during the extrusion process, the GPC analysis was first performed on r-PLA and then compared with the extruded one (e-r-PLA).

As shown in Table 3, PLA’s molecular weight decreases after extrusion due to the previous processing phase, which may affect its thermostability (Table 3, entry 1 vs. entry 2). Conversely, in the presence of 2.5 or 5 wt% of biochar filler, a pronounced effect of stabilization was observed (Table 3, entries 4, 5 vs. entry 2). Therefore, the filler can notably reduce the adverse effect of the considered PLA processing on molecular mass. The observed stabilization of PLA by biochar can be attributed to scavenging traces of water from melts, which reduces the hydrolysis of polyester molecules, as previously reported for carbon fillers [6].

On the contrary, by reducing the filler at 1 wt%, biochar does not have any influence on the PLA stability (Table 3, entry 3).

### 3.3. Thermal Properties

**Figure 2:**
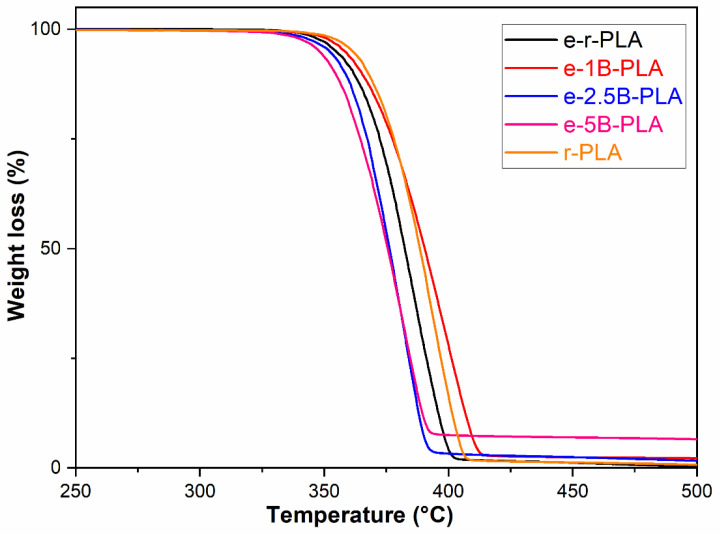
TGA profiles of PLA-based composites: e-r-PLA (black line), e-1B-PLA (red line), e-2.5B-PLA (blue line), e-5B-PLA (magenta line), and r-PLA (yellow line).

Thermogravimetric analysis was conducted on neat r-PLA and its composites with different percentages of B. As shown in Figure 2, the thermal decomposition began at a lower temperature after extrusion (Figure 2, e-r-PLA), pointing out the negative effect of the melt processing on r-PLA.

The same effect also occurs with 2.5 and 5wt % biochar, but contrary to what is reported in the literature [9], the temperature does not decrease proportionately with biochar percentage increases, possibly due to the different composition of residual inorganic ashes. In contrast to the 74% of calcium (as calcium carbonate), much higher than that reported in the literature, the inorganic composition of B, as determined by ICP-MS, shows a limited amount of potassium (17%) that is reported to be active in PLA degradation [34,35]. As calcium carbonate is a recognized stabilizing filler in many polymer matrices [36], it can compensate for the degradation caused by potassium.

Therefore, the TGA profile is stable in the presence of 2.5 and 5wt % of B.

However, when the filler is reduced to 1 wt%, the thermal degradation is similar to neat r-PLA due to the lesser ashes affecting PLA degradation pathways. Therefore, the stabilization effect of calcium carbonate prevails, slowing down the thermal degradation of PLA polymers.

DSC analysis was used to determine the effect of biochar additive on the thermal transitions of r-PLA composites. The first heating cycle was performed to eliminate the thermal history of the polymeric material [37]. As a result, the thermal behavior of the second heating cycle was considered for discussion. The glass transition temperature (T_g_), melting temperature (T_m_), cold crystallization temperature (T_cc_), experimental melting enthalpy (ΔH_m_), and crystallinity degree (X_c_) of neat and reinforced PLA composites are reported in Table 4.

**Figure 3:**
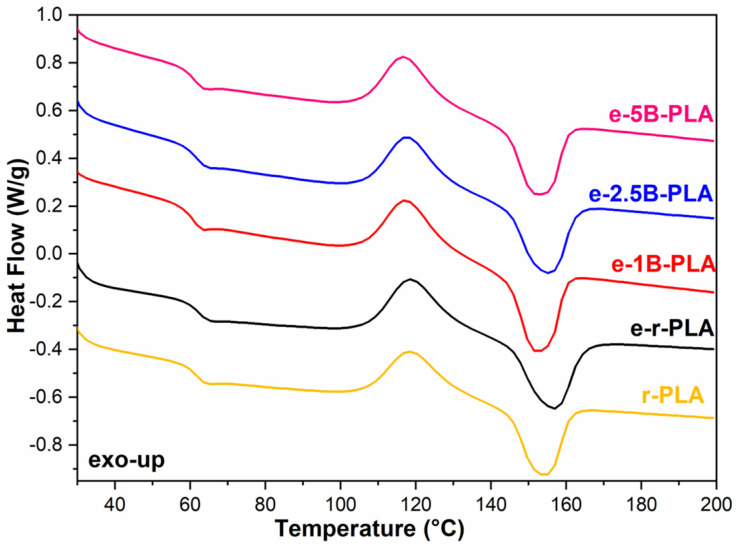
DSC thermogram from the second heating cycle of the PLA-based composites.

PLA composites did not exhibit any visible effects from biochar addition at 1, 2.5, or 5 wt% on the T_g_; further, a slight shift in T_CC_ was observed only when 5 wt% B was added (Figure 3).

Concerning the crystallinity degree, the additional melt processing step does not influence the PLA samples, which seem to be essentially amorphous (Table 3) regardless of the presence or the absence of filler in the polymer matrix.

### 3.4. Processability and Mechanical Properties

**Figure 4:**
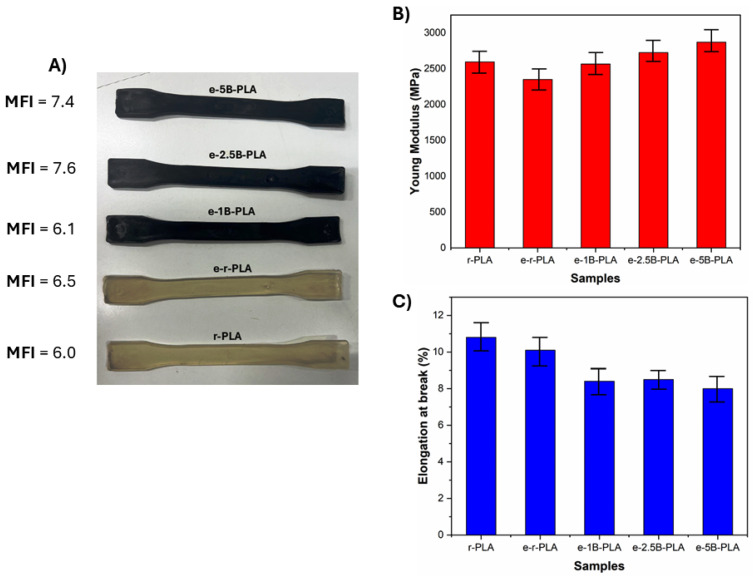
(**A**) PLA and reinforced PLA specimens, (**B**) Young modulus, (**C**) Elongation at break of the PLA-based composite specimens.

The processability of the composites so obtained is a fundamental step of the recycling approach, being crucial to the possibility of manufacturing new products. Therefore, the melt flow index (MFI) of the composites was evaluated. As reported in Figure 4 A the values of MFI show a slight variation between 6.0 and 7.4, which does not affect the injection molding process.

The mechanical properties of PLA composite specimens with different percentages of B were studied and compared with r-PLA after extrusion.

The incorporation of biochar powder into composites can increase the elastic modulus for e-1B-PLA, e-2.5B-PLA, and e-5B-PLA by 12.5%, 17%, and 20%, respectively, compared to e-r-PLA, although elongation at break decreases by up to 21% (Figure 4C). As a result of the intermolecular forces between B particles and PLA molecules, the elastic modulus of the composite material increased, possibly due to biochar particles restricting the mobility of PLA molecules [39].

To evaluate the stability of the r-PLA composites, aging treatments were performed on the specimens in a climatic chamber for 23 h and 90 h at λ 340 nm and 63 °C, simulating the common storage industrial conditions of about 1 and 3, 5 months, respectively.

In order to determine the effect of the process and aging treatment on the composites, the specimens from the starting r-PLA and the melt-extruded samples were analyzed using DCS analysis, considering the thermal behavior of the first heating. As reported in Table 5, in most cases, the T_g_ decreases during the aging treatment, with a less pronounced response in the presence of 1 wt% of biochar.

**Figure 5:**
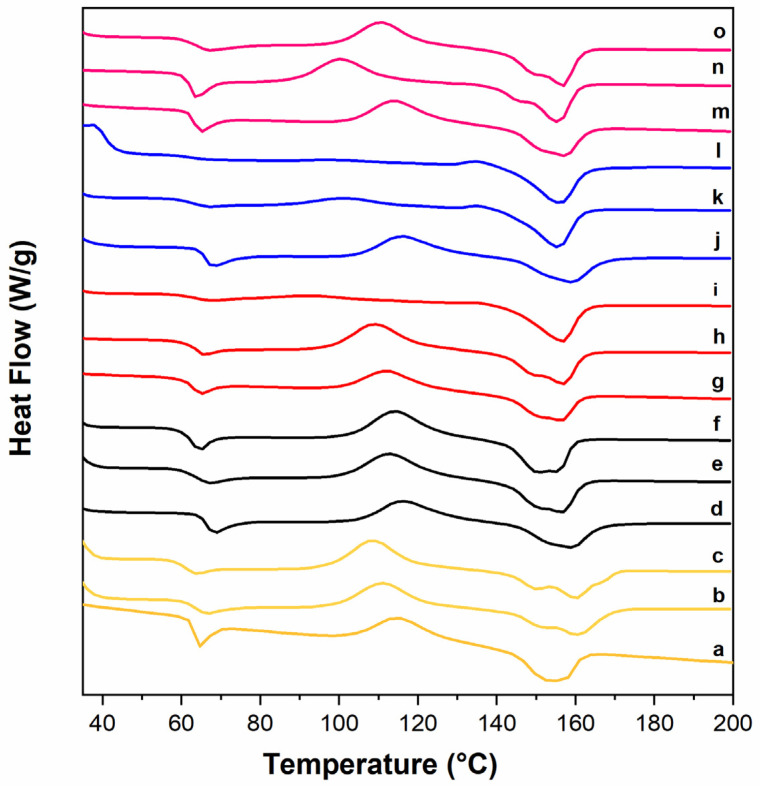
DSC thermograms of r-PLA (Yellow), e-r-PLA (Black), e-1B-PLA (Red), e-2.5-PLA (Blue), and e-5B-PLA (dark pink) as obtained after extrusion (a,d,g,j,m), after 23 h (b,e,h,k,n), and 90 h (c,f,i,l,o) of aging treatment under UV irradiation at 60 °C.

Moreover, an interesting effect appears in the presence of 1 and even more of 2.5 wt% of biochar. The T_g_, characteristic of the amorphous phase, as well as the cold crystallization peak, is evident in both the starting materials but progressively becomes less pronounced after 23 h aging (Figure 5h,k) and almost disappears after 90 h aging (Figure 5i,l). At the same time, the melting temperature becomes more defined, and the crystallization degree substantially increases going from an amorphous sample as freshly prepared to a crystalline PLA up to 30% of crystalline degree in the presence of 2.5 wt% of B (Table 5).

This evidences a nucleation effect on PLA crystallization that is significant for 1 wt% of B but more pronounced for 2.5 wt%, which shows an increase in crystallinity of 25% after 23 h and a rise of 30% after 90 h of aging.

Conversely, increasing biochar concentration to 5 wt% has negligible effects on crystallization, probably due to filler aggregation in the polymer matrix.

Furthermore, the molecular weight shows an interesting variability during the aging process. While for r-PLA, the molecular weight decreased after 23 h and more after 90 h, a different tendency was observed for e-r-PLA and e-1B-PLA. As a result, after 23 h of aging, an increased molecular weight was recorded, possibly due to the double effect of chain scission and crosslinking polymerization induced by UV [40]. As previously reported, the chain scission of polymer chains under irradiation is accompanied by the crosslinking process, mainly when higher doses are applied, due to the generation of radical species. Prolonged exposure to UV and at 60 °C can induce these phenomena.

More interesting is the result obtained after 90 h of aging treatment. As reported in Table 6, after the initial stabilization effect on the molecular weight after processing PLA/biochar composites, the presence of biochar preserves the PLA from further degradation. Specifically, while the starting pellet r-PLA results in a continuous degradation during the aging treatment as well as e-r-PLA after 90 h, biochar has a significant stabilizing effect, raising the molecular weights by 50.7 kDa and 48.5 kDa for e-1B-PLA and e-2.5B-PLA, after starting at 41.5 kDa and 47.3 kDa, respectively.

Additionally, a higher percentage of B, although ineffective in stabilizing the molecular weight after 23 h, preserves PLA from further degradation after 90 h aging, keeping constant the Mn (42.0 kDA vs. 41.5 kDa).

The stabilization effect of B in the polymer matrix under the aging treatment is also evidenced by the TGA measurements.

**Figure 6:**
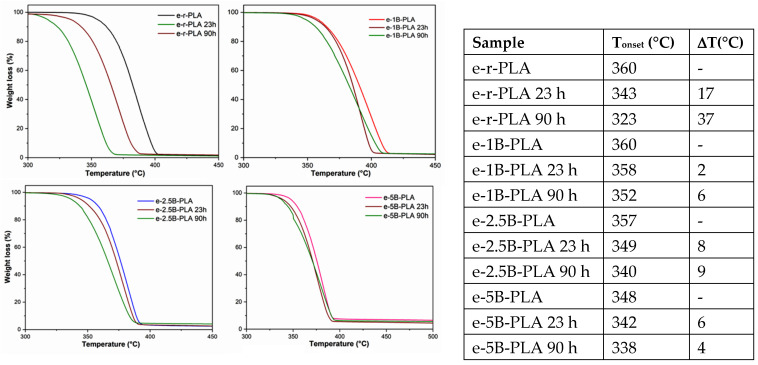
TGA profiles and T_onset_ of e-r-PLA, e-1B-PLA, e-2.5B-PLA, and e-5B-PLA as obtained in black, red, blue, and dark pink, respectively, and of the corresponding aged samples after 23 h (wine line) and 90 h (green line).

As shown in Figure 6, the degradation onset temperature (T_onset_) evaluated on e-r-PLA dramatically changed during the aging, going from 360 °C of the starting material to 323 °C after 90 h irradiation with a significant reduction of 40 °C. Conversely, by adding B, in all the cases, the T_onset_ shows a less pronounced decrease between 2–9 °C, further confirming biochar’s stabilization effect in the polymer matrix. The observed stabilization induced by biochar filler can be explained by the significant amount of calcium carbonate that, as previously reported [8], can act as a safeguard against UV light penetration by creating a protective barrier.

## 4. Conclusions

This is the first study of the effect of biochar on regenerated PLA in which the circular economy is respected from the choice of the polymer matrix as the regenerated one to the filler as a renewable source. The composites were produced with 1, 2.5, and 5 wt% by melt extrusion and then analyzed by GPC, DSC, and TGA.

The results show the ability of biochar to provide a composite by extrusion with higher stability during tprocessing, providing a significant stabilization of the molecular weight when 2.5 and 5 wt% of filler are used. The observed stabilization of PLA by biochar can be attributed to scavenging traces of water from melts, which reduces the hydrolysis of polyester molecules.

TGA measurement evidenced that, although the thermal decomposition of extruded r-PLA began at a lower temperature than the raw material (r-PLA), the presence of biochar, due to its high percentage of calcium carbonate, can slow down the thermal degradation of PLA polymers.

To estimate the processability of the composites, the melt flow index was evaluated by using the D1238 ASTM procedure, giving values ranging from 6.0 for the starting material to 7.4 for e-5B-PLA and demonstrating that the presence of biochar does not affect the injection molding process.

The mechanical properties of PLA composite specimens with different percentages of biochar were studied and compared with r-PLA after extrusion. The results show that the incorporation of biochar powder into composites can increase the elastic modulus by up to 20% more than that of raw material.

To evaluate the stability of the r-PLA composites, aging treatments were performed on the specimens in a climatic chamber for 23 h and 90 h, simulating the common storage industrial conditions of about 1 and 3.5 months, respectively.

Based on the DSC analysis, biochar’s nucleation effects are already evident for 1 wt% of B but are more significant for 2.5 wt% of B. After 23 h of aging, the crystallinity increases by 25% and by 30% after 90 h.

Further, TGA analysis indicated a significant thermostability in the presence of biochar, and GPC analyses confirmed that biochar stabilized the polymer matrix, maintaining a high or constant molecular weight after 90 h due to the presence of the consistent amount of Calcium carbonate acting as a safeguard against UV light penetration by creating a protective barrier.

## Figures and Tables

**Table 1:** Characterization of biomass (P) and corresponding biochar (B).

Samples	Yield	C	H	N	O	Fixed Carbon	Volatiles	Ash	pH	pHpzc	BET *
	wt%	wt% daf				wt% db					m^2^/g
*P*	-	48.67	5.91	0.18	45.24	16.6	79.9	3.5	-	-	-
*B*	32.2	78.29	2.85	0.61	18.25	61.52	27.51	10.96	9.8	8.2	60

* Surface analysis was evaluated by Brunauer–Emmett–Teller theory.

**Table 2:** ICP-MS analysis of biomass (P) and corresponding biochar (B).

		P	e% ^a^	B	e% ^a^
Na	mg/Kg	175	7	326	12
Mg		670	7	1627	15
Al		315	0	654	3
K		2511	16	8397	21
Ca		11,000	8	27,330	9
Fe		790	1	1785	17

^a^ Percentage error is given in brackets.

**Table 3:** Number average molecular weight (*M*n), weight average molecular weight (*M*w), and polydispersity index (PDI) as evaluated by GPC curves for r-PLA and extruded compounds. The evaluated variance is of ±3 kDa.

Entry	Sample	M_n_ (kDa)	MW (kDa)	PDI
1	r-PLA	59.3	113.1	1.9
2	e-r-PLA	42.7	101.6	2.4
3	e-1B-PLA	41.5	101.2	2.4
4	e-2.5B-PLA	47.3	101	2.1
5	e-5B-PLA	47.1	97.7	2.1

**Table 4:** DSC analysis results of PLA-based composites.

Samples	T_g_ (°C)	T_m_ (°C)	T_CC_ (°C)	ΔH_cc_ (J/g)	ΔH_m_ (J/g)	χ_c_ (%)
r-PLA	58.6 ± 0.2	153.4 ± 0.3	118.4 ± 0.2	16.2 ± 0.4	20.2 ± 0.3	4.3 ± 0.1
e-r-PLA	58.7 ± 0.1	155.5 ± 0.4	118.2 ± 0.1	17.3 ± 0.2	23.2 ± 0.2	6.3 ± 0.0
e-1B-PLA	58.3 ± 0.3	151.3 ± 0.2	117.2 ± 0.3	18.6 ± 0.3	22.9 ± 0.3	4.3 ± 0.2
e-2.5B-PLA	58.3 ± 0.2	154.7 ± 0.4	117.4 ± 0.4	17.6 ± 0.2	22.0 ± 0.2	4.8 ± 0.0
e-5B-PLA	57.9 ± 0.1	151.7 ± 0.2	116.6 ± 0.5	17.2 ± 0.4	20.7 ± 0.2	3.9 ± 0.2

The degree of crystallization was estimated based on the melting enthalpy for a fully crystalline alpha-form PLLA sample as evaluated by Garlotta (93.7 J/g) [38].

**Table 5:** DSC analysis results of PLA-based composites under aging treatment.

Samples	T_g_ (°C)	T_m_ (°C)	T_CC_ (°C)	ΔH_cc_ (J/g)	ΔH_m_ (J/g)	χ_c_ (%)
r-PLA	61.7 ± 0.3	152.4 ± 0.5	114.7 ± 0.4	25.5 ± 0.5	20.8 ± 0.6	-
r-PLA (23 h aged)	60.9 ± 0.3	159.8 ± 0.3	111 ± 0.6	21.8 ± 0.6	26.9 ± 0.4	5.4 ± 0.2
r-PLA (90 h aged)	58.0 ± 0.2	159.9 ± 0.4	108.5 ± 0.3	25.4 ± 0.4	27.5 ± 0.5	2.2 ± 0.1
e-r-PLA	64.4 ± 0.1	158 ± 0.4	116.1 ± 0.2	17.9 ± 0.2	21.9 ± 0.3	4.3 ± 0.1
e-r-PLA (23 h aged)	60.8 ± 0.2	155.8 ± 0.5	112.8 ± 0.7	22.1 ± 0.5	26.1 ± 0.4	4.3 ± 0.1
e-r-PLA (90 h aged)	60.1 ± 0.2	154.0 ± 0.5	114.1 ± 0.4	24.6 ± 0.3	25.8 ± 0.2	-
e-1B-PLA	60.8 ± 0.2	155.9 ± 0.4	111.8 ± 0.3	16.3 ± 0.2	20.4 ± 0.4	4.4 ± 0.2
e-1B-PLA (23 h aged)	61.2 ± 0.1	156.4 ± 0.3	108.8 ± 0.2	21.0 ± 0.5	25.1 ± 0.5	4.4 ± 0.0
e-1B-PLA (90 h aged)	59.3 ± 0.3	156.5 ± 0.6	/	/	26.6 ± 0.1	29
e-2.5B-PLA	64.3 ± 0.1	157.9 ± 0.1	115.7 ± 0.3	19.9 ± 0.4	19.5 ± 0.2	-
e-2.5B-PLA (23 h aged)	58.7 ± 0.3	154.7 ± 0.2	101.3 ± 0.2	4.7 ± 0.3	27.3 ± 0.2	25
e-2.5B-PLA (90 h aged)	58.6 ± 0.3	155.1 ± 0.2	/	/	27.4 ± 0.1	30
e-5B-PLA	61.6 ± 0.2	157.0 ± 0.3	113.7 ± 0.5	17.9 ± 0.5	24.7 ± 0.3	7.6 ± 0.2
e-5B-PLA (23 h aged)	55.9 ± 0.4	156.7 ± 0.4	103.5 ± 0.2	18.2 ± 0.3	24.3 ± 0.4	6.9 ± 0.0
e-5B-PLA (90 h aged)	59.3 ± 0.3	155.0 ± 0.2	110.4 ± 0.6	20.4 ± 0.3	27.7 ± 0.4	8.2 ± 0.1

The degree of crystallization was estimated based on the melting enthalpy for a fully crystalline alpha-form PLLA sample as evaluated by Garlotta (93.7 J/g) [38].

**Table 6:** Number average molecular weight (*M*n), weight average molecular weight (*M*w), and polydispersity index (PDI) as evaluated by GPC curves for r-PLA and extruded compound after aging treatment. The evaluated variance is of ±3 kDa.

Entry	Sample	M_n_ (kDa)	MW (kDa)	PDI
1	r-PLA	59.3	113.1	1.9
2	r-PLA (23 h aged)	54.9	99.5	1.8
3	r-PLA (90 h aged)	49.3	94.3	1.9
4	e-r-PLA	42.7	101.6	2.4
5	e-r-PLA (23 h aged)	47.3	97.9	2.1
6	e-r-PLA (90 h aged)	43.2	89.8	2.1
7	e-1B-PLA	41.5	101.2	1.8
8	e-1B-PLA (23 h aged)	51.7	94.5	2.1
9	e-1B-PLA (90 h aged)	50.7	89.6	1.8
10	e-2.5B-PLA	47.3	101	2.1
11	e-2.5B-PLA (23 h aged)	41.1	85.7	2.1
12	e-2.5B-PLA (90 h aged)	48.5	85.5	1.8
13	e-5B-PLA	47.1	97.7	2.1
14	e-5B-PLA (23 h aged)	42.0	79.0	1.9
15	e-5B-PLA (90 h aged)	41.5	80.5	1.9

## Data Availability

The original contributions presented in the study are included in the article, further inquiries can be directed to the corresponding author.
